# A Novel Quantitative Hemolytic Assay Coupled with Restriction Fragment Length Polymorphisms Analysis Enabled Early Diagnosis of Atypical Hemolytic Uremic Syndrome and Identified Unique Predisposing Mutations in Japan

**DOI:** 10.1371/journal.pone.0124655

**Published:** 2015-05-07

**Authors:** Yoko Yoshida, Toshiyuki Miyata, Masanori Matsumoto, Hiroko Shirotani-Ikejima, Yumiko Uchida, Yoshifumi Ohyama, Tetsuro Kokubo, Yoshihiro Fujimura

**Affiliations:** 1 Department of Blood Transfusion Medicine, Nara Medical University, Kashihara, Japan; 2 Department of Molecular Pathogenesis, National Cerebral and Cardiovascular Center, Suita, Japan; 3 Molecular and Cellular Biology Laboratory, Graduate School of Medical Life Science, Yokohama City University, Yokohama, Japan; The Hospital for Sick Children and The University of Toronto, CANADA

## Abstract

For thrombotic microangiopathies (TMAs), the diagnosis of atypical hemolytic uremic syndrome (aHUS) is made by ruling out Shiga toxin-producing *Escherichia coli* (STEC)-associated HUS and ADAMTS13 activity-deficient thrombotic thrombocytopenic purpura (TTP), often using the exclusion criteria for secondary TMAs. Nowadays, assays for ADAMTS13 activity and evaluation for STEC infection can be performed within a few hours. However, a confident diagnosis of aHUS often requires comprehensive gene analysis of the alternative complement activation pathway, which usually takes at least several weeks. However, predisposing genetic abnormalities are only identified in approximately 70% of aHUS. To facilitate the diagnosis of complement-mediated aHUS, we describe a quantitative hemolytic assay using sheep red blood cells (RBCs) and human citrated plasma, spiked with or without a novel inhibitory anti-complement factor H (CFH) monoclonal antibody. Among 45 aHUS patients in Japan, 24% (11/45) had moderate-to-severe (≥50%) hemolysis, whereas the remaining 76% (34/45) patients had mild or no hemolysis (<50%). The former group is largely attributed to CFH-related abnormalities, and the latter group has C3-p.I1157T mutations (16/34), which were identified by restriction fragment length polymorphism (RFLP) analysis. Thus, a quantitative hemolytic assay coupled with RFLP analysis enabled the early diagnosis of complement-mediated aHUS in 60% (27/45) of patients in Japan within a week of presentation. We hypothesize that this novel quantitative hemolytic assay would be more useful in a Caucasian population, who may have a higher proportion of CFH mutations than Japanese patients.

## Introduction

Thrombotic thrombocytopenic purpura (TTP) with predominantly neurological involvement and hemolytic uremic syndrome (HUS) with predominately renal failure are both life-threatening systemic diseases that are often clinically indistinguishable. They are categorized as thrombotic microangiopathies (TMAs) [1, 2]. It is now well documented that TTP is caused by deficiency of ADAMTS13 (a disintegrin-like and metalloproteinase with thrombospondin type 1 motifs 13) activity, either because of genetic abnormalities or acquired autoantibodies [3, 4]. On the other hand, more than 90% of HUS cases are associated with Shiga toxin-producing *Escherichia coli* (STEC) infection, termed STEC-HUS or typical HUS. The remaining 10% or so, which does not involve STEC infection, is called atypical HUS (aHUS) [5].

Most cases of aHUS are caused by uncontrolled complement activation due to genetic abnormalities in the alternative pathway, including complement factor H (CFH), complement factor I (CFI), membrane cofactor protein (MCP), thrombomodulin (THBD), complement component C3 (C3), and complement factor B (CFB) [6]. Acquired autoantibodies against CFH can also mediate aHUS; they are frequently associated with homozygous gene deletion of CFH-related (CFHR) proteins 1 and 3 [7–9]. More recently, recessive mutations in diacylglycerol kinase ε, a protein kinase C inhibitor, were also shown to cause aHUS. Diacylglycerol kinase ε normally blocks signaling of arachidonic acid-containing diacylglycerols involved in platelet activation [10]. However, unlike ADAMTS13 deficiency in TTP and STEC infection in typical HUS, making a diagnosis of aHUS is not easy. In fact, comprehensive gene analysis takes at least several weeks, and can only detect genetic abnormalities in approximately 70% of the patients with aHUS [11].

In 2004, Sanchez-Corral et al. [12] introduced a qualitative hemolytic assay using sheep red blood cells (RBCs). This assay is based on the principle that in normal individuals, exogenous human CFH, via its glycosaminoglycan-binding domains in the C-terminal portion, binds to the sialic acid-rich surface of sheep RBCs, to which C3b binds and is then proteolytically inactivated by CFI. Ultimately, this results in inhibition of hemolysis. In contrast, mutant CFH may not readily bind to the surface of sheep RBCs, which results in an inability to block hemolysis associated with C3b generated from spontaneous hydrolysis, followed by the formation of membrane attack complex. In 2014, Roumenina et al. [13] reported on a modified hemolytic assay using serum or EDTA plasma. However, depending on how they were prepared and preserved, serum specimens often give inconsistent results in the hemolytic assays. In addition, it is not possible to measure ADAMTS13 activity using EDTA plasma because the enzyme is a divalent cation-dependent metalloproteinase [4]. Thus, existing hemolytic assays are qualitative in nature.

In this study, we have developed a quantitative hemolytic assay using sheep RBCs with human citrated plasma spiked with or without the novel inhibitory anti-CFH murine monoclonal antibody (mAb) O72. This was followed by genetic analysis of 45 aHUS patients in Japan. We found that this novel quantitative hemolytic assay plus RFLP analysis can be used for the early diagnosis of aHUS patients in Japan. Subsequent gene analysis identified a unique predisposing mutation accumulated in the Kansai district of Japan. Thus, although the number of tested patients is still small, the genetic abnormalities of aHUS patients in Japan appear to be different from those in Western countries.

## Materials and Methods

### Ethics statement

The study protocol was approved by the Ethics Committees of Nara Medical University Hospital and National Cerebral and Cardiovascular Center, and complied with the principles expressed in the Declaration of Helsinki. All patients were given written informed consent to participate in this study. All animal studies were approved by the Institutional Review Board of Nara Medical University. To sacrifice animals we used cervical dislocation, and all efforts were made to minimize suffering.

### Patients

Since 1998, our laboratory at Nara Medical University has enrolled patients with suspected TMA based on clinical characteristics across Japan [14]. As of the end of 2013, we established a registry of 1,214 patients with TMA. Patient plasmas were sent to our laboratory, and firstly ADAMTS13 activity was measured. Of these 1,214 patients, severe deficiency of ADAMTS13 activity (<5% of normal) was found in 467 patients, of which 52 had congenital TTP and 415 had acquired TTP. The primary diagnosis of aHUS was made based on Japanese criteria for aHUS [15]: microangiopathic hemolytic anemia (hemoglobin <10 g/dl), thrombocytopenia (platelet count of <150×10^9^/l), and acute renal failure (pediatric patients: serum creatinine 1.5-fold higher than the age- and gender-specific Japanese Society for Pediatric Nephrology reference values [16]; adult patients: meeting diagnostic criteria for acute kidney injury (AKI)) with no severe ADAMTS13 activity deficiency or STEC infection. In this study, however, the diagnosis of aHUS was also made according to the exclusion criteria used in the UK [17] and Spain [18], where patients with organ or hematopoietic stem cell transplantation, systemic lupus erythematosus and pregnancy-associated are excluded. As a result, there were 77 aHUS patients in our cohort as of the end of 2013, of whom 45 patients belonged to 40 families that were analyzed by the novel hemolytic assay, and then followed by genetic analysis. Among them, 43 patients were tested using freshly prepared citrated plasmas, but in two patients (2P1 and 3G1) the sera stored at -80^°^C were only available for analysis. Plasma or serum levels of CFH and C3 in 43 of 45 patients were described in S1 Table. C3 level was determined by immune-nephelometry (SRL, Inc., Japan). CFH level was measured by Laurell’s immunoelectrophoresis using rabbit anti-CFH serum prepared in our laboratory. A value of 100% of plasma CFH antigen level was defined by the amount in the pooled normal plasmas, prepared from a total of 20 healthy individuals (10 males and 10 females).

### Purification of CFH from human plasma

Anti-CFH rabbit polyclonal antibody (pAb) was a gift from Professor Emeritus Teizo Fujita of Fukushima Medical University. Twenty milligrams of anti-CFH IgG pAb were coupled to 2 g of CNBr-activated Sepharose 4B (GE Healthcare Bio-Sciences AB, Uppsala, Sweden) according to the manufacturer’s instructions. Ten milliliters of fresh frozen plasma were diluted three times with starting buffer consisting of 50 mM Tris-buffered saline (pH 7.4) containing 20 mM ε-aminocaproic acid, 2 mM phenylmethylsulfonyl fluoride, 2 mM benzamidine-HCl (BZ), 5 mM EDTA, and 0.02% sodium azide (NaN_3_), and then applied to the immunoadsorbent column. The bound proteins were eluted with 0.1 M glycine-HCl (pH 2.7) containing 2 mM BZ and 0.02% NaN_3_. The eluted protein fractions were immediately neutralized and dialyzed overnight at 4°C against 20 mM Tris-HCl buffer (pH 8) containing 2 mM BZ and 0.05% NaN_3_. After dialysis, the samples were applied to a Mono Q 5/50 GL column (GE Healthcare Bio-Sciences AB) and the bound proteins were eluted with a linear salt gradient of 0 to 1 M NaCl. Purified plasma CFH was eluted as a single major peak at 0.3 M NaCl. These fractions were pooled, concentrated with Aquacide II (Calbiochem, San Diego, CA, USA), dialyzed against Tris-buffered saline, and then stored in aliquots at −80°C until use.

### Preparation of anti-CFH monoclonal antibodies (mAbs)

BALB/c mice were injected intraperitoneally with 50 μg of purified human CFH mixed with Freund’s complete adjuvant (Difco Laboratories, Detroit, MI, USA) every two weeks for a total of four times. After immunization, the procedure for anti-CFH mAb production, which consisted of cell fusion, culture, and cloning, was performed according to a well-established method [19]. Six different clones that produce anti-CFH IgG mAbs were obtained, and their specificities were confirmed by Western blot analysis. The immunoglobulin subclass of the anti-CFH mAbs was determined using the Mouse/Rat Monoclonal Antibody Isotyping Test Kit (AbD Serotec, Kidlington, UK).

### Construction of expression plasmids for recombinant CFH and CFH domains

To produce recombinant human CFH in budding yeast, various lengths of *CFH* fragments with a C-terminal Pk (V5) tag were inserted into a p416GAL1 vector [20] using the Sequence and Ligation Independent Cloning method unless otherwise noted [21]. In addition to full-length CFH, four truncated forms of CFH were generated as follows: short consensus repeat (SCR) 1–5 (plasmid pM7315), SCR6–10 (pM7316), SCR11–17 (pM7317) and SCR18–20 (pM7318). In addition, SCR18–20 was divided into three peptides: SCR18 and 19 (pM7336), SCR 19 and 20 (pM7340), and SCR 18 and 20 (pM7341). Full-length *CFH* (3693 bp) or these truncated forms were PCR-amplified from sequence-verified human *CFH* cDNA from the Mammalian Gene Collection (Clone ID: 40148771) with the corresponding primers, as well as a Pk (V5) tag fragment (132 bp) that was amplified by PCR from pM4376 [22]. As for the construction of pM7336, the fragment was prepared from pM7335 using the restriction enzyme SacI and XhoI, and was inserted in the p426GAL1 vector [20]. In pM7341, the expression plasmid for SCR18 and 20 was obtained in a two-step PCR reaction. The first PCR reaction was performed with two primer pairs, after which the fragments were connected by the second PCR (primer TK12150-TK12151). Detailed information on each recombinant CFH and its domain expression plasmid and primer are shown in S2 Table.

### Expression of recombinant CFH and its fragments and Western blot analysis

*Saccharomyces cerevisiae* BY4741 (Euroscarf, Frankfurt, Germany) was transformed with each of the expression plasmids for recombinant CFH and its fragments. Transformed *Saccharomyces cerevisiae* cells were cultured in 20 ml of SD medium (0.17% yeast nitrogen base without amino acids and ammonium sulfate, 0.5% ammonium sulfate, 2% glucose) or SG medium (0.17% yeast nitrogen base without amino acids and ammonium sulfate, 0.5% ammonium sulfate, 4% galactose) at 30°C until the mid-logarithmic growth phase. The treatment of cell extracts and immunoblot analysis was performed as previously described [23]. Anti-CFH mAb O72 (1:1000) and horseradish peroxidase (HRP)-labeled anti-mouse IgG (1:2000; #sc-2005, Santa Cruz Biotechnology, Dallas, TX, USA) were used for the detection of recombinant CFH and its fragments. Samples were developed using enhanced chemiluminescence (ECL) substrate (Thermo Fisher Scientific, Waltham, MA, USA, and Merck Millipore, Darmstadt, Germany) and detected on an LAS 4000 image analyzer (GE Healthcare, Buckinghamshire, England).

### Hemolytic assay using citrated human plasma and sheep RBCs

Sheep RBCs were purchased from Japan Ram Co (Fukuyama, Japan). Hemolytic assays using sheep RBCs were performed according to the method of Sanchez-Correal et al and Roumenina et al [12, 13], except that throughout our study we used citrated plasma unless otherwise noted.

Briefly, we diluted 5–60 μl of normal or tested citrated plasma or serum into 100 μl with AP-CFTD buffer (2.5 mM barbital, 1.5 mM sodium barbital, 144 mM NaCl, 7 mM MgCl_2_, and 10 mM EGTA, pH 7.2–7.4). Each sample of diluted plasma (100 μl) was then mixed with 100 μl of sheep RBC suspensions prepared with AP-CFTD buffer to a final concentration of 2.5×10^6^ cells/μl. The mixture was further incubated at 37°C for 30 min. After incubation, the reaction was quenched by the addition of 1 ml of VBS-EDTA buffer (2.5 mM barbital, 1.5 mM sodium barbital and 144 mM NaCl, and 50 mM EDTA, pH 7.4). The mixtures were then centrifuged at 800 g at 4°C for 10 min, and the absorbance of the supernatant was measured at 414 nm. Plasma, diluted with AP-CFTD buffer containing 2 mM EDTA was treated in the same manner and used as a blank. In this study, 100% hemolysis was defined as the absorbance at 414 nm obtained with 20 μl of normal citrated plasma spiked with mAb O72 (200 μg/ml). The percentage of hemolysis in the patients was calculated as follows: the absorbance of the 20 μl plasma samples with the corresponding blank subtracted was divided by the absorbance of 20 μl normal plasma spiked with mAb O72. The hemolytic assay was performed in duplicate, and the mean value was used for calculation or shown as the result of the hemolytic assay. To determine the normal ranges of hemolysis, we tested hemolysis on freshly prepared citrated plasmas from 20 healthy individuals (10 males and 10 females).

In some experiments, to assess the inhibitory effect of anti-CFH IgG mAbs, we incubated 20 μl of normal plasma mixed with each anti-CFH mAb or control mouse IgG at a final concentration of 50, 100, 200, 300, or 400 μg IgG/ml at room temperature for 30 min. Subsequently, these mixtures were diluted to 100 μl with AP-CFTD buffer. The diluted samples were mixed with 100 μl of sheep RBC suspensions (final concentration, 1×10^6^ cells/μl) and these mixtures were assayed as described above.

### Determination of anti-CFH autoantibody status with Western blot

The presence of anti-CFH autoantibodies in each patient’s plasma or serum was evaluated using Western blot analysis, as previously described [24]. Briefly, purified CFH was loaded on a 5% SDS-PAGE gel under non-reducing conditions, and then transferred to a polyvinylidene difluoride membrane. After blocking with 5% skim milk, the membrane was cut into strips of 0.5 cm in width. Each strip was incubated with a 1:100 dilution of a patient plasma or serum sample, followed by HRP-labeled goat anti-human IgG antibody (1:1000; #074–1006, KPL, Gaithersburg, MD, USA). Bound HRP-labeled antibodies were visualized using an enhanced ECL substrate (Western Lightning Plus ECL, PerkinElmer, Waltham, MA, USA).

### Determination of anti-CFH autoantibody titer with ELISA

Plasma or serum samples of five patients with anti-CFH autoantibodies detected by Western blot were further analyzed by a CFH IgG ELISA kit (Abnova, Taipei, Taiwan). All procedures were performed according to the manufacturer’s instructions. Samples of patient specimens were analyzed in duplicate and diluted if needed. Various concentrations of CFH IgG (0, 3.9, 7.8, 15.6, 31.3, 62.5, 125, 250 Arbitrary Unit (AU)/ml) were prepared by supplied 10,000 AU/ml stock solution. Antibody titers were calculated by the standard curve composed of above concentrations of CFH IgG (3.9–250 AU/ml).

### Determination of CFH-related proteins using Western blot

Tested plasmas or sera were collected before plasma therapy. Two microliters of plasmas or sera were electrophoresed on a 12% SDS-PAGE gel, transferred to a polyvinylidene difluoride membrane, and blocked with 5% skim milk. Blotted proteins were incubated with a monoclonal mouse anti-human CFHR1 antibody (1 μg/ml, #MAB4247, R&D Systems, Minneapolis, MN, USA) or a polyclonal rabbit anti-human CFHR3 antibody (1:1500, #16583-1-AP, Proteintech, Chicago, IL, USA). After three washes, the blots were incubated with a HRP-labeled anti-mouse or rabbit IgG antibody (1:10000 and 1:20000, respectively; #074–1806, #074–1506, KPL). Bound antibodies were visualized using the aforementioned methods.

### Genetic analysis and structure model

Genetic analysis was performed at the Department of Molecular Pathogenesis, the National Cerebral and Cardiovascular Center as previously described [24]. Genomic DNA was extracted from peripheral blood leukocytes of patients and their family members. The coding exons and the intronic flanking regions of *CFH* (NM 000186.3), *C3* (NM 000064.2), *MCP* (NM 002389.4), *CFI* (NM 000204.3), *CFB* (NM 001710.5), and *THBD* (NM 000361.2) were amplified using PCR and sequenced. The adenine of the ATG translation initiation start site was designated as the +1 position and the initial Met was denoted as +1. Of the 45 patients, W1 was sequenced at other institution [25].

The mutations, which have previously been reported as the cause of aHUS, were described as ‘predisposing mutation’. The rare mutations were described as ‘potentially predisposing mutation’. RFLP analysis was performed to detect C3-p.I1157T mutation as previously described [24]. Amplified DNA fragments were digested with the restriction enzyme SspI (New England Biolabs, Ipswich, MA, USA) and the products were electrophoresed to determine the genotype based on the cleaved bands.

The crystal structures of the complexes of C3b/CFH-SCR1-4 (ID: 2WII) and C3d/CFH-SCR19-20 (ID: 3OXU) were retrieved from the Protein Data Bank (http://www.rcsb.org/pdb/home/home.do) [26, 27]. Molecular graphic imaging was generated by using the PyMOL molecular visualization system (Schrödinger, Portland, OR).

## Results

### Characterization of the six anti-CFH mAbs

**Fig 1:**
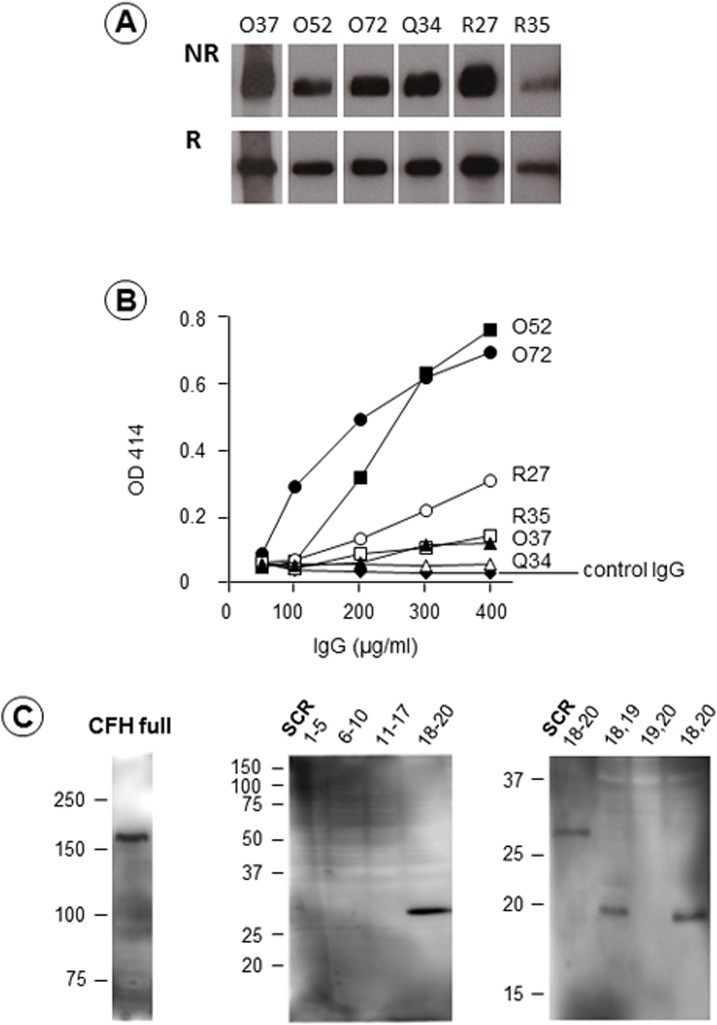
Production of six anti-complement factor H (CFH) murine monoclonal antibodies (mAbs), and their effects on lysis of sheep red blood cells (RBCs). **(A) Western blot analysis.** All six anti-CFH mAbs reacted with purified human CFH on Western blot under both non-reducing (NR) and reducing (R) conditions. **(B) Effects of six anti-CFH mAbs on lysis of sheep RBCs.** Twenty microliters of normal plasmas spiked with anti-CFH mAbs were incubated with sheep RBCs at a final concentration of 50 μg/ml to 400 μg/ml. Two plasma samples, treated with anti-CFH mAb O52 and O72, induced strong hemolysis in a dose-dependent manner. Three mAbs (R27, R35, and O37) resulted in slightly enhanced hemolysis. As for mAb Q34, appreciable hemolysis was not detected, similar to control IgG. **(C) Epitope analysis of mAb O72 using recombinant CFH expressed by yeast.** The mAb O72 reacted with full length CFH (left) and short consensus repeat (SCR) 18–20 (middle). Moreover, the right panel shows that mAb O72 reacted with peptide of SCR18−19, and SCR18 and 20, but not SCR19−20, indicating that the epitope of mAb O72 resided in SCR18.

Six murine anti-CFH mAbs, termed O37, O52, O72, Q34, R27, and R35, reacted with CFH purified from plasma with Western blot under both non-reducing and reducing conditions (Fig 1A). Immunoglobulin isotyping revealed that five mAbs (O37, O72, Q34, R27, and R35) were IgG1κ and one mAb (O52) was IgG2aκ. Then, we evaluated the ability of these six anti-CFH IgG mAbs to induce hemolysis through inhibition of CFH function, as originally reported by Józsi et al and Strobel et al [28, 29].

As shown in Fig 1B, two anti-CFH IgG mAbs (O52 and O72) induced strong hemolysis, almost indistinguishably from each other. Three other mAbs (R27, R35, and O37) induced slightly enhanced hemolysis, but the mAb Q34 did not induce any appreciable hemolysis.

Next, we determined the epitope of the anti-CFH mAb O72 using recombinant CFH fragments expressed by yeast with Western blot. As shown in Fig 1C, the anti-CFH mAb O72 reacted not only to full-length recombinant human CFH, but also to SCR18–20. However, mAb O72 did not react with SCR1–5, SCR6–10, or SCR11–17. To narrow down mAb O72 epitope, we prepared three short peptides within SCR18–19, SCR19–20, and SCR18 and 20. The Western blot clearly indicated that mAb O72 reacted with both peptide of SCR18–19, and SCR18 and 20, but not with SCR19–20, indicating that mAb O72 epitope resided in SCR18. Similarly, we determined the mAb O52 epitope to be in SCR16 (data not shown).

### Optimal quantitative hemolytic assay

Since normal plasma spiked with anti-CFH mAb O72 consistently induced enhanced hemolysis in the sheep RBCs assay through blocking the C-terminus region of CFH from binding to sialic acid on the RBCs surface, spiked normal plasma was used as a positive control in the hemolytic assays throughout this study. Two groups of investigators have previously indicated that the hemolytic assays could be performed using serum, EDTA plasma, or citrated plasma [12, 13]. A majority of patients with TMA often need an assay of ADAMTS13 activity concurrently for ruling out TTP, which can only be stably measured using citrated plasma. Thus, we evaluated conditions for a quantitative hemolytic assay using citrated plasma as follows.

**Fig 2:**
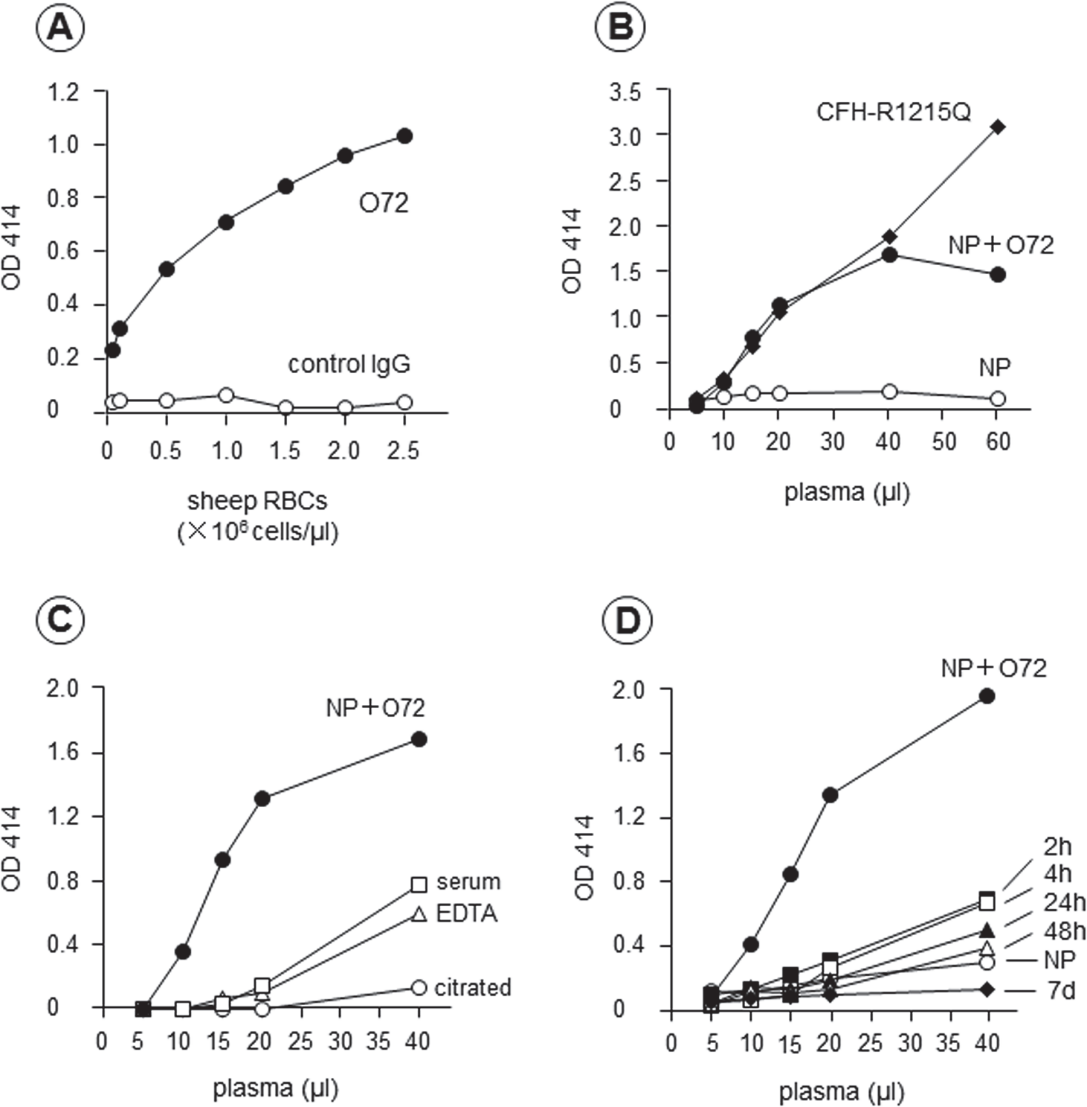
Optimization of the hemolytic assay: sheep red blood cells (RBCs) concentration, anti-complement factor H (CFH) monoclonal antibody (mAb) O72, and blood specimen type. **(A) Evaluation of the optimal sheep RBC count.** Various counts of sheep RBCs were incubated with 20 μl of normal plasma spiked with anti-CFH mAb O72 (200 μg IgG/ml, NP + O72) or control IgG. Maximum hemolysis was observed at the sheep RBC count of 2.5×10^6^/μl; thus, all subsequent experiments were performed using this sheep RBC count. **(B) Comparison of hemolysis induced by mAb O72 and CFH mutant protein.** The degree of hemolysis caused by normal plasma spiked with mAb O72 or plasma from a patient with a CFH-p.R1215Q mutation was quite comparable up to 40 μl. **(C) Screening of optimal blood specimen type.** Three different specimens (serum, EDTA plasma, and citrated plasma) from one healthy donor were analyzed using the hemolytic assay. Citrated normal plasma spiked with mAb O72 was used as a positive control. In serum and EDTA plasma, mild hemolysis of sheep RBCs was detected at 40μl. No appreciable hemolysis was observed at any volume of citrated plasma. **(D) Evaluation of the influence of the coagulation time interval on hemolytic reaction.** Serum obtained 2 and 4 hours after blood collection showed mildly enhanced hemolysis at 40 μl. This mild hemolysis was lower in serum obtained 24 and 48 hours prior. No appreciable hemolysis was detected in serum obtained 7 days prior.

First, the optimal sheep RBC count was evaluated as follows: 20 μl of normal plasma spiked with anti-CFH mAb O72 (final concentration, 200 μg IgG/ml) or control mouse IgG was incubated for 30 min at room temperature, and then diluted to 100 μl with AP-CFTD buffer. To this mixture, we added 100 μl of sheep RBC suspensions at various cell counts ranging from 0.1×10^6^ to 2.5×10^6^ cells/μl, which was then further incubated at 37°C for 30 min. After incubation, the reaction was quenched by adding 1 ml of VBS-EDTA buffer. As shown in Fig 2A, the maximum difference in hemolysis between normal plasma samples spiked with mAb O72 and control IgG mAb was observed at the sheep RBC count of 2.5×10^6^ /μl.

Second, the degree of hemolysis induced by normal plasma samples spiked with mAb O72 was compared to that of plasma samples from a patient with a CFH-p.R1215Q mutation (described below) by increasing the volume of plasma added. As shown in Fig 2B, normal plasma alone at the volume of 5–60 μl did not induce appreciable hemolysis, but plasma samples spiked with mAb O72 or plasma samples with a CFH-p.R1215Q mutation showed enhanced hemolysis in a dose-dependent manner, which was almost indistinguishable up to a volume of 40 μl.

Third, hemolytic assay performed using three different blood specimens from a healthy individual were compared: 1) serum prepared from a blood collection tube, 2) EDTA plasma, and 3) citrated plasma. As shown in Fig 2C, appreciable hemolysis was not noted up to 20 μl in all specimens, while normal citrated plasma spiked with mAb O72 showed enhanced hemolysis at that volume. On the other hand, normal citrated plasma alone showed minimal hemolysis at 40 μl, but both serum and EDTA plasma at this volume showed enhanced hemolysis without any additives.

Fourth, we evaluated the coagulation time interval for generating serum after blood collection on the hemolytic assay, since thrombin or other proteases generated during coagulation may affect hemolysis via complement activation. For this purpose, 50 ml of whole blood was drawn from a healthy individual, which was separated into 5 ml aliquots in sterile glass test tubes. The blood was kept at room temperature for 2, 4, 24, and 48 hours, and 7 days. At each time interval, blood was centrifuged at 800 g for 15 min at 4°C to separate serum, which was stored at −80°C until use. The hemolytic assay was performed using these freezing-thawed serum samples. As shown in Fig 2D, serum samples obtained at the interval of 2 and 4 hours showed slightly enhanced hemolysis at a volume of 40 μl. However, serum obtained at 7 days no longer showed enhanced hemolysis, even in the presence of mAb O72.

**Fig 3:**
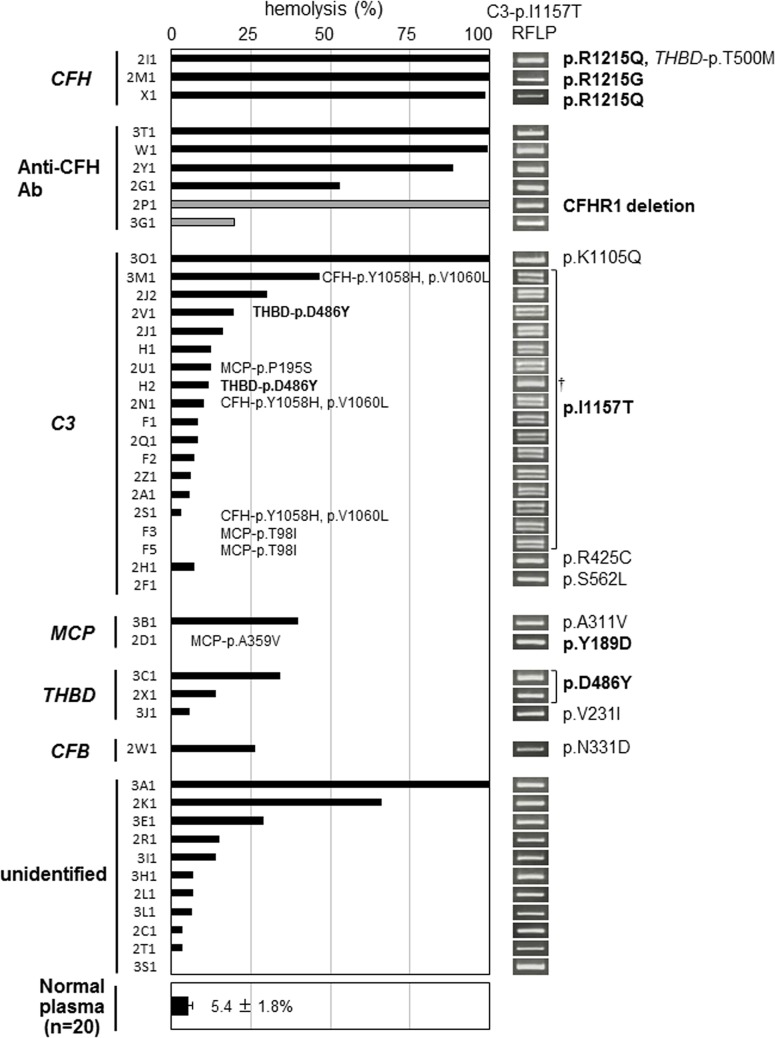
Relationship between hemolytic activity and complement abnormalities in 45 patients with atypical hemolytic uremic syndrome (aHUS). The results of the hemolytic assay for 45 aHUS patients are shown according to the predisposing (bold type) or potentially predisposing mutations and/or the acquired abnormalities. The patients carrying two mutations were classified according to the predisposing mutation, and the other mutations were described alongside the predisposing mutations or black bars. Two patients (2V1 and H2) were categorized into the C3 group, although they had two predisposing mutations (C3-p.I1157T and THBD-p.D486Y). In two patients (2P1 and 3G1), the hemolysis induced by serum was represented by gray bars. The normal range of hemolysis (5.4±1.8%: mean ± standard deviation) obtained from 20 healthy individuals (10 males and 10 females) is shown at the bottom of Fig 3. The results of the restriction fragment length polymorphism (RFLP) analysis of C3-p.I1157T are summarized to the right of the hemolytic assay results. Regarding the RFLP analysis of C3-p.I1157T (c.3470T>C), the heterozygous mutation (T/C) showed two bands (278 bp, 314 bp) and the homozygous mutation (C/C) showed one band (314 bp). † H2 had a homozygous mutation in C3-p.I1157T.

Taken together, sheep RBCs at a final concentration of 2.5×10^6^ cells/μl and citrated plasma were chosen for the assay. Furthermore, 100% hemolysis was defined as the absorbance at optimal density (OD) 414 nm obtained with 20 μl of normal citrated plasma spiked with mAb O72 (200 μg/ml). Under these conditions, freshly prepared citrated plasmas from 20 healthy individuals were tested using the hemolytic assay, and showed the degree of hemolysis as 5.4±1.8% (mean ± standard deviation) (Fig 3).

### Quantitative hemolytic assay plus genetic analysis for aHUS

In total, 45 aHUS patients underwent genetic analysis for six candidate genes (*CFH*, *C3*, *MCP*, *THBD*, *CFB*, *CFI*). The results of the hemolytic assay and complement abnormalities are shown in Fig 3.

The degree of hemolysis was classified into four categories based on the percentage of hemolysis as follows: severe hemolysis: >75% hemolysis (9 patients), moderate hemolysis: 50–75% hemolysis (2 patients), mild hemolysis: 25–50% (6 patients), and no apparent hemolysis: <25% (28 patients).

#### 1.Severe hemolysis: >75%

There were nine unrelated patients (2I1, 2M1, X1, 3T1, W1, 2Y1, 2P1, 3O1, and 3A1) with more than 75% hemolysis, of whom seven patients (excluding 3O1 and 3A1) had CFH-related abnormalities (Fig 3). Among them, three patients (2I1, 2M1 and X1) carried a predisposing mutation, either p.R1215Q or p.R1215G in the SCR20 domain of CFH. Moreover, anti-CFH autoantibodies were detected in four patients (3T1, W1, 2Y1 and 2P1). The antibody titers in three patients except for 2P1 were 46,784, 13,700, and 360 AU/mL, respectively (S3 Table). Patient 2P1 showed deficiency of CFHR1 protein in serum [30], and 2Y1 displayed low but detectable levels of CFHR1 according to Western blot. Both 3T1 and W1 did not have any deletions in CFHR1 and CFHR3. Of the two remaining patients, Patient 3A1 had no mutations in the six candidate genes, but the degree of hemolysis decreased from 100% to 30% during plasma exchange treatment and to 7% after initiation of eculizumab therapy.

**Fig 4:**
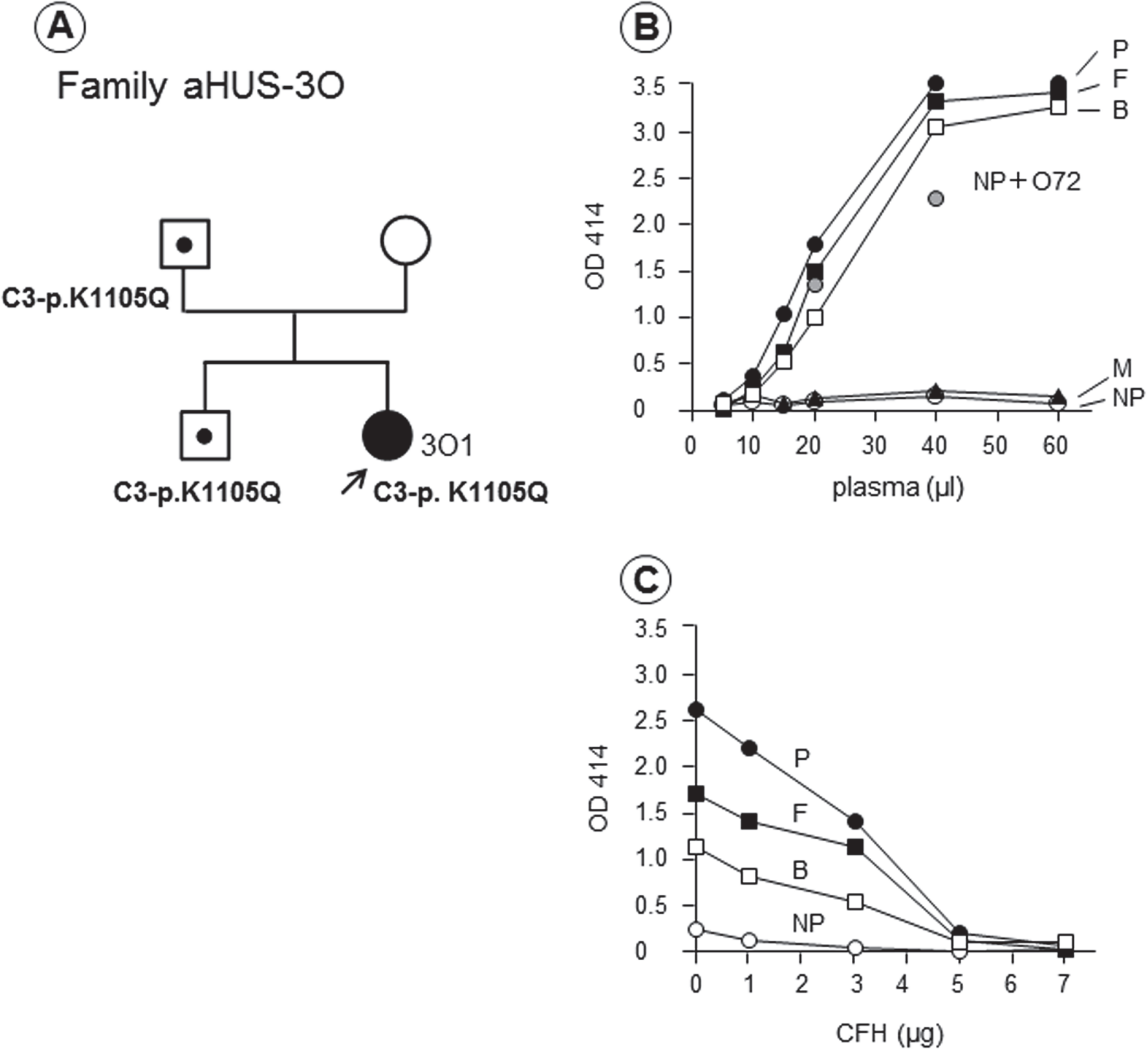
Family 3O with a C3-p.K1105Q mutation. (A) Patient 3O1 (female) developed atypical hemolytic uremic syndrome (aHUS) at the age of five months. (B) In the hemolytic assay, plasma samples from three family members (patient: P, father: F, and brother: B) induced strong hemolysis of sheep red blood cells (RBCs), but her mother (M) had no appreciable hemolysis, with results similar to normal plasma (NP). (C) Lysis of sheep RBCs detected in the three family members was corrected by the addition of complement factor H (CFH) in a dose-dependent manner. Genetic analysis showed that the patient and her father and brother, whose plasma clearly showed hemolysis, carried a potentially predisposing mutation p.K1105Q in C3, not in CFH.

In a female Patient 3O1, a heterozygous mutation at p.K1105Q within the thioester-containing domain (TED) of C3 was identified, but no mutations in CFH were identified. As shown in Fig 4A, her father and brother, who have the same mutation but were asymptomatic, showed similar severe hemolytic reactions (Fig 4B). Hemolysis observed in these three family members was corrected by the addition of purified CFH (Fig 4C). The patient’s mother did not have an enhanced hemolytic reaction or the mutation. Anti-CFH autoantibodies were negative in all members of this family tested.

**Fig 5:**
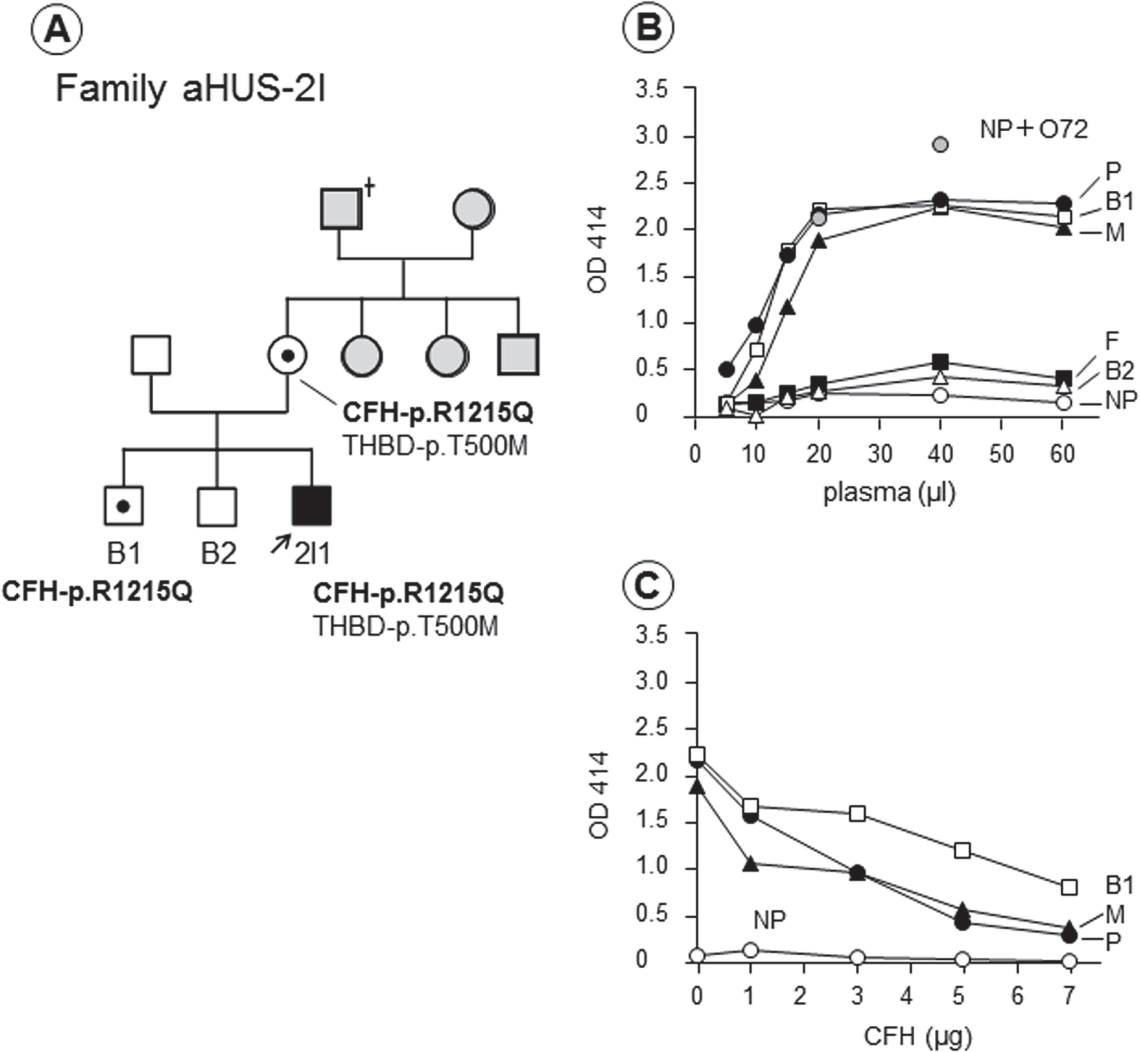
Family 2I with a CFH-p.R1215Q mutation. (A) Patient 2I1 (male) had episodes of atypical hemolytic uremic syndrome (aHUS) at the age of 28 and 29. Gray squares or circles indicate individuals who were not analyzed in this study. (B) The hemolytic assay showed that plasma from the patient (P), his mother (M), and one brother (B1) induced severe hemolysis, whereas samples from his father (F) and the other brother (B2) showed no appreciable hemolysis. (C) The hemolysis detected in the three family members was corrected by the addition of purified complement factor H (CFH). Genetic analysis showed that the patient, his mother, and brother 1 carried a CFH-p.R1215Q mutation, but his mother and brother 1 have never had the episodes of aHUS.

A potentially important and interesting finding from the hemolytic assay was observed in the family of Patient 2I1, who had a heterozygous p.R1215Q mutation in CFH (Fig 5A). As shown in Fig 5B, strongly enhanced hemolysis was detected in this patient, his mother, and his older brother (brother 1) in the hemolytic assay. Dose-dependent inhibition of enhanced hemolysis was seen when purified CFH was added to the reaction mixtures for these three individuals (Fig 5C), who all carried the same p.R1215Q mutation in CFH. One potentially predisposing mutation, THBD-p.T500M, was also identified in the patient and his mother (Fig 5A). Interestingly, the patient’s mother (now 61 years old) and brother 1 (now 34 years old) have never had any episodes of TMA. We also identified that unaffected X1’s father with the CFH-p.R1215Q mutation had hemolysis greater than 50% in the hemolytic assay.

#### 2.Moderate hemolysis: 50–75%

Two aHUS patients, 2G1 and 2K1, had 50–75% hemolysis, but we were unable to identify any predisposing mutations through the analysis of the six candidate genes (Fig 3). In Patient 2G1, anti-CFH autoantibodies were detected by both Western blot and CFH IgG ELISA (4,813 AU/mL) (S3 Table), although she had no deficiency in CFHR1 and CFHR3 proteins.

**Fig 6:**
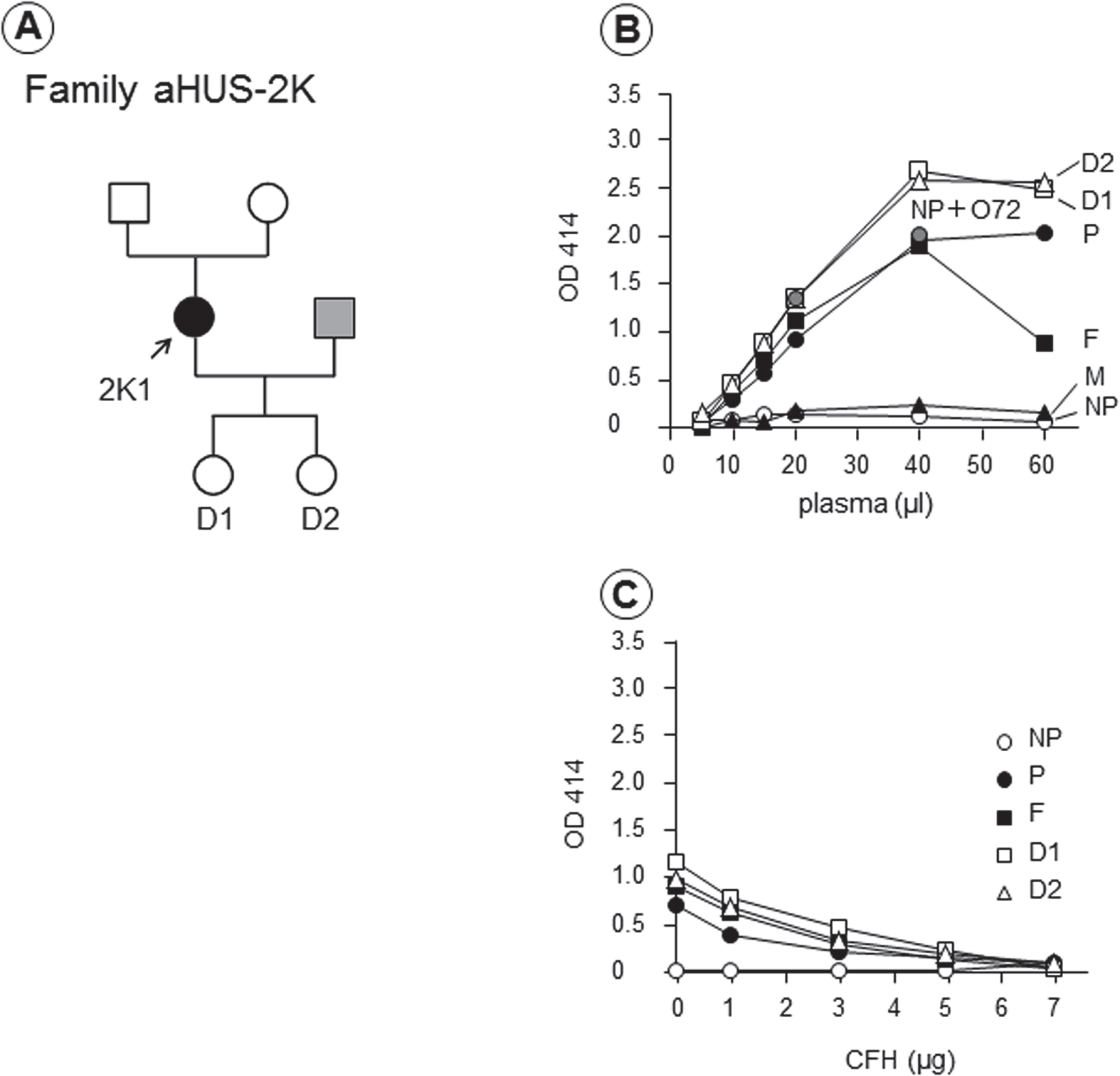
Family 2K with no predisposing or potentially predisposing mutations. (A) Patient 2K1 (female) developed atypical hemolytic uremic syndrome (aHUS) at the age of 35. Her husband (gray square) was not analyzed in this study. (B) The hemolytic assay showed that plasma from the patient (P), her father (F), and two daughters (D1 and D2) induced severe hemolysis, which was not observed in her mother (M). (C) The enhanced hemolysis detected in these four family members was corrected by the addition of purified complement factor H (CFH). However, no predisposing or potentially predisposing mutations were detected in Patient 2K1.

On the other hand, no autoantibodies were identified in Patient 2K1 (Fig 6A). Samples from her parents and two children were also analyzed using the hemolytic assay. As shown in Fig 6B, her father and both children showed severely enhanced hemolysis, but her mother showed no appreciable hemolysis. The enhanced hemolysis detected in these four family members was corrected by the addition of purified CFH (Fig 6C). However, none of the family members had anti-CFH autoantibodies or CFH deficiency. These results suggest that the family of Patient 2K1 might have a genetic abnormality involved in the complement regulation system, inherited from her father.

#### 3. Mild hemolysis: 25–50%

Six patients (3M1, 2J2, 3B1, 3C1, 2W1, and 3E1) were in this category. Three patients had predisposing mutations. Patients 3M1 and 2J2 had a p.I1157T mutation in the TED of C3 (described below) and Patient 3C1 had a p.D486Y mutation in the serine-threonine-rich region of THBD. Furthermore, two other patients (3B1 and 2W1) had potentially predisposing mutations: p.A311V in the transmembrane region of MCP, and p.N331D in the von Willebrand type A domain of CFB, respectively. Only one patient (3E1) had no abnormalities in the six candidate genes and the CFHR1 and CFHR3 proteins based on Western blot.

#### 4. No apparent hemolysis: <25%

There were 28 patients in this category, of whom 16 had C3 mutations. Surprisingly, among these 16 patients, 14 had the same predisposing mutation, p.I1157T in the TED of C3. The remaining two patients (2H1 and 2F1) had potentially predisposing mutations in C3, p.R425C and p.S562L, respectively. Two patients (2D1 and 2X1) had predisposing mutations in two transmembrane proteins. One had a p.Y189D mutation in the SCR3 domain of MCP, and the other had a p.D486Y mutation in THBD. Patient 3J1 had a potentially predisposing mutation, p.V231I in THBD. Curiously, Patient 3G1 having anti-CFH autoantibody, but without deletion in CFHR1 and CFHR3 proteins, was also categorized in this group. This finding was quite different from other patients having anti-CFH autoantibodies. Further, the antibody titer in 3G1 (5,995 AU/mL) determined by quantitative ELISA was higher than that of 2Y1 (360 AU/mL) and 2G1 (4,813 AU/mL) (S3 Table). Thus, the discrepancy between the degrees of hemolysis and antibody titer might be attributable to the epitope specificity of anti-CFH autoantibodies. On the other hand, Patient 3G1 had low C3 level (30 mg/mL) (S1 Table), which might contribute to a low degree of hemolysis.

**Fig 7:**
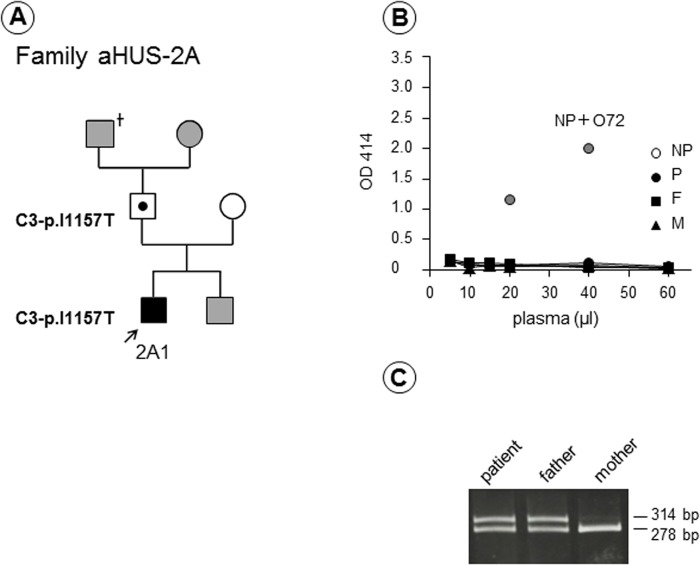
Family 2A with a C3-p.I1157T mutation. (A) Patient 2A1 (male) had six bouts of atypical hemolytic uremic syndrome (aHUS), at the age of 9, 15, 18, 22 and 29 (twice). Gray squares or circle indicate individuals who were not analyzed in this study. (B) The hemolytic assay showed that neither patient (P) nor his parents (father: F and mother: M) had appreciable hemolysis. However, Patient 2A1 and his father carried the same predisposing mutation p.I1157T in C3, confirmed by restriction fragment length polymorphism (RFLP) analysis (C) and direct DNA sequencing.

As a consequence, the most frequent genetic abnormality associated with congenital aHUS in Japan, p.I1157T in C3, was unable to be detected by the hemolytic assay. In Fig 7A, we showed the family of Patient 2A carrying a heterozygous C3-p.I1157T mutation as a representative example. Neither the patient nor his family members had enhanced hemolysis (Fig 7B), but the RFLP analysis of C3-p.I1157T clearly indicated that the patient and his asymptomatic father carried the same heterozygous predisposing mutation (Fig 7C), which was also confirmed by direct DNA sequencing. The results of the RFLP analysis in all 45 patients are shown alongside in the hemolytic assay results (Fig 3). These results clearly indicated whether the patient had a heterozygous or homozygous mutation in C3-p.I1157T or not, and 16 carriers were easily diagnosed by the RFLP analysis. Thus, in total a high frequency of a single C3 gene mutation (36%, 16/45) was identified in our aHUS cohort.

Most interestingly, a geographical distribution of gene mutations of aHUS patients showed that the patients carrying the C3-p.I1157T mutation were only found in an extremely restricted area (Kansai district including Mie [31], Nara, Kyoto, and Osaka) and not found in other areas of West Japan (S1 Fig).

## Discussion

Rapid differential diagnosis of TMA is needed in clinical practice to select the optimal therapeutic approach. Evaluations of ADAMTS13 activity and STEC infection are often necessary, both of them usually can be done within a few hours. Further, comprehensive genetic analysis has been proposed as being able to consistently diagnose aHUS in approximately 70% of patients, but it usually takes at least several weeks, resulting in almost no clinical utility during emergencies. Since a relatively high proportion (approximately 20–30%) of mutations occurs in CFH among Caucasian aHUS patients [5, 11], the usefulness of a qualitative hemolytic assay using sheep RBCs has been postulated [12, 13]. However, it appears that the hemolytic assay has not been commonly used in many laboratories. This may be due to unstable viability of sheep RBCs over long-term storage at 4°C, unreal 100% hemolysis of sheep RBCs exposed to distilled water, and the type of blood specimens used, such as serum, EDTA plasma, or citrated plasma. Here, we have shown that serum is not a good specimen for the hemolytic assay, and EDTA plasma cannot be used in the ADAMTS13 activity assay.

In this study, we have successfully generated a novel murine anti-CFH mAb O72, which induces severe hemolysis at 200 μg/ml (final concentration) when spiked into an assay mixture of normal human plasma and sheep RBCs. The mAb O72 epitope resides in SCR18, which could explain its ability to block CFH binding to the sialic acid-rich cell surface of sheep RBCs. Interestingly, the degree of hemolysis induced by normal plasma spiked with mAb O72 is almost the same as that mediated by plasma from a patient with a heterozygous CFH-p.R1215Q mutation. In both these plasma samples, enhanced hemolysis caused by the presence of nonfunctional CFH was corrected by the addition of purified CFH in a dose-dependent manner, and totally eliminated at high concentrations. By using the novel quantitative hemolytic assay, we have retrospectively analyzed 45 aHUS patients in our cohort. The aHUS is an extremely rare disease, and so far only one hundred patients have been diagnosed across Japan. We are expecting to find merely 10–15 new patients every year, and therefore the prospective analysis is difficult to perform at the moment. This is why we performed retrospective analysis in this study.

Based on the quantitative hemolytic assay, 11 of 45 aHUS patients in Japan had more than 50% (moderate-to-severe) hemolysis, in whom the following gene mutations or abnormalities were identified: two patients (2I1 and X1) with CFH-p.R1215Q, one patient (2M1) with CFH-p.R1215G, five patients (3T1, W1, 2Y1, 2G1 and 2P1) with anti-CFH Ab, one patient (3O1) with C3-p.K1105Q, and two patients (3A1 and 2K1) without any gene mutations. Severe hemolysis was also identified in three asymptomatic family members (Patient 2I’s mother and brother 1, and Patient X1’s father) with a heterozygous CFH-p.R1215Q mutation. Presently, we are unable to address whether they are asymptomatic carriers or latent patients.

An interesting family (aHUS-2K) was identified by the hemolytic assay as shown in Fig 6. In this family, plasmas of the patient, her father, and two daughters caused severe hemolysis, while the mother’s plasma did not cause hemolysis in this assay. However, predisposing mutations were not identified in Patient 2K1, and anti-CFH autoantibodies were negative in all members of this family tested. Roumenina et al. [13] reported that the presence of a hybrid CFH/CFHR1 gene caused appreciable hemolysis of sheep RBCs. Thus, this patient might have a hybrid CFH/CFHR1 gene, or hitherto unrecognized genetic defect, inherited from the paternal side. Notably, without this novel hemolytic assay, evidence of complement-associated aHUS in this patient would have been difficult to obtain. The C3-p.K1105Q mutation in Patient 3O1 is a unique exception, which will be discussed later.

The remaining 34 aHUS patients had less than 50% (almost none-to-mild) hemolytic reactions, in whom the following predisposing gene mutations were identified: 16 patients with C3-p.I1157T, two patients (3C1 and 2X1) with THBD-p.D.486Y, one patient (2D1) with MCP-p.Y189D and nine patients with no mutations and no CFH autoantibodies. Moreover, one patient (3G1) had anti-CFH autoantibodies.

This is the first report that a patient with the C3 mutation, C3-p.K1105Q shows severe hemolytic activity (Figs 3 and 4), because usefulness of the hemolytic assay has been mostly referred to functional defects of CFH in previous publications [12, 13]. The C3-p.K1105Q mutation is located in the TED of C3 as shown in S2A Fig. A structural model of the complex of C3d with CFH-SCR19-20 showed that the C3-p.K1105Q mutation was positioned at the contact interface between C3d and CFH-SCR19, suggesting the defect of C3d binding to CFH-SCR19 (S2C Fig). In contrast, the p.I1157T mutation identified in 16 aHUS patients with low (less than 50%) hemolytic activity was positioned at the contact interface between C3b and CFH-SCR4, suggesting the defect of C3b binding to CFH-SCR1-4 (S2B Fig). Based on these differences, we assume that C3-p.K1105Q mutant with defective binding to CFH-SCR19 could induce the severe hemolysis.

We also identified interesting patients of 3B1 with MCP mutation and 3C1 with THBD mutation with mild hemolysis (25–50%), because both of these transmembrane proteins do not directly affect *in vitro* hemolysis. Thus, presently the mechanism of mild hemolysis shown in these plasma samples left unaddressed.

We found that the frequency of C3 mutation in aHUS patients in Japan (42%, 19/45) was much higher than that in other aHUS cohorts in Western countries [32–34] (Table 1). Moreover, Schramm et al. recently reported the frequency of C3 mutation in the French, Italian, UK, and USA aHUS cohorts to be 11.4%, 4.6%, 6.1% and 4.5%, respectively [35]. As shown in S1 Fig, the patients with C3-p.I1157T mutation were only found in the Kansai district, West Japan [31] and not found in other areas of Japan, suggesting a reflection of ‘founder effect’. Now, we have started to perform a cohort study of aHUS in East Japan.

**Table 1:** Comparison of genetic or acquired abnormalities among Western countries and Japan.

	Frequency			
Genetic or acquired	Noris et al	Maga et al	Fremeaux-Bacchi et al	Yoshida et al
abnormalities	(n = 273)	(n = 144)	(n = 214)	(n = 45)
	Italy	USA	France	Japan
*CFH*	24%	27%	28%	7%
anti-CFH Ab	3%	ND	7%	13%
*C3*	4%	2%	8%	42%
*MCP*	7%	5%	9%	5%
*THBD*	5%	3%	0%	7%
*CFB*	0.4% (n = 1)	4%	2%	2%
*CFI*	4%	8%	8%	0%
unidentified	~30%	54%	34%	24%

The frequency of each genetic or acquired abnormality in our aHUS cohort in Japan was compared to that in three other cohorts (Italy [32], USA [33], France [34]). The data reported by Noris et al. included 47 secondary aHUS patients. Sixty-percent of aHUS patients in our cohort were enrolled from West Japan. In the data of Japan, both predisposing and potentially predisposing mutations were counted. Although two patients (2V1 and H2) had two predisposing mutations (C3-p.I1157T and THBD-p.D486Y), they were counted as C3 group.

CFH: complement factor H, Ab: antibody, C3: complement component C3, MCP: membrane cofactor protein, THBD: thrombomodulin, CFB: complement factor B, CFI: complement factor I, ND: no data

In summary, we report that 24% (11/45) of aHUS patients in Japan have moderate-to-severe (≥50%) hemolysis, whereas the remaining 76% (34/45) patients had mild or almost no hemolysis (<50%). In the former group, this was largely attributed to CFH abnormalities, whereas in the latter group, 16 patients carried C3-p.I1157T mutation, which was identified by a simple RFLP analysis. We assume that this novel quantitative hemolytic assay would be more useful for the diagnosis of aHUS in Caucasians, who have a higher proportion of CFH mutations.

## Supporting Information

S1 Fig Geographical distribution of patients with atypical hemolytic uremic syndrome (aHUS) in Japan. Each aHUS patient is described as a black or red circle. Sixteen patients with C3-p.I1157T mutation shown by red circles were found only in the Kansai district, including Mie, Nara, Kyoto, and Osaka prefectures. Interestingly, patients carrying C3-p.I1157T mutation were not found in other areas of West Japan, which may indicate a reflection of ‘founder effect’.
(TIF)

S2 Fig Locations of the C3-p.I1157T and C3-p.K1105Q mutations in the C3b-complement factor H (CFH)-SCR1-4 complex and the C3d-CFH-SCR19-20 complex. **(A) A structural model of the complex of C3b and short consensus repeat (SCR) 1–4 of CFH (ID: 2WII).** The α and β chains of C3b are shown in cyan and blue, respectively and the SCR1-4 domains are depicted with gray, red, orange, and magenta, respectively. The I1157 and K1105 residues shown by the grey-labeled side chains are located in the thioester-containing domain (TED) in C3b. **(B) Close-up view of the region around the contact interface between C3b and SCR1-4.** The p.I1157T mutation identified in 16 patients with low (less than 50%) hemolytic activity, but not the p.K1105Q mutation identified in a patient with high (100%) hemolytic activity, is positioned at the interface between C3b and SCR1-4 and would interfere the C3b binding to CFH-SCR1-4. **(C) A structural model of the complex of C3d and SCR19-20 of CFH (ID: 3OXU).** C3d, SCR19, and SCR20 are depicted with cyan, blue, and yellow, respectively. The p.K1105Q mutation, but not the p.I1157T mutation, is positioned at the interface between C3d and SCR19-20, and would interfere the C3b binding to CFH-SCR19-20. Diagram was generated with the PyMOL molecular visualization system.
(TIF)

S1 Table The levels of C3 and complement factor H (CFH) protein in 43 of 45 patients with atypical hemolytic uremic syndrome (aHUS). C3 level was determined by immune-nephelometry (SRL, Inc., Japan), and CFH level was measured by Laurell’s immunoelectrophoresis using rabbit anti-CFH serum prepared in our laboratory. The levels of C3 and CFH in 43 aHUS patients and the mean ± standard deviation of these patients and normal plasma from 20 healthy individuals were described. CFH: complement factor H, C3: complement component C3, NP: normal plasma, ND: not determined
(TIF)

S2 Table Plasmids and primers for expression of recombinant complement factor H (CFH) and its domains. SCR: short consensus repeat, CFH: complement factor H, Pk tag: GKPIPNPLLGLDST sequence, aa: amino acids.
(TIF)

S3 Table Characteristics of five of six patients with anti-complement factor H (CFH) autoantibodies according to ELISA. Determination of CFH autoantibody titer was performed by CFH-IgG ELISA kit (Abnova). Antibody titer was calculated according to the manufacturer’s protocol by using standard curve. AU: arbitrary unit, ND: not determined.
(TIF)
