# Correction: A Novel Quantitative Hemolytic Assay Coupled with Restriction Fragment Length Polymorphisms Analysis Enabled Early Diagnosis of Atypical Hemolytic Uremic Syndrome and Identified Unique Predisposing Mutations in Japan

**DOI:** 10.1371/journal.pone.0178015

**Published:** 2017-05-16

**Authors:** Yoko Yoshida, Toshiyuki Miyata, Masanori Matsumoto, Hiroko Shirotani-Ikejima, Yumiko Uchida, Yoshifumi Ohyama, Tetsuro Kokubo, Yoshihiro Fujimura

There are two errors in the “Hemolytic assay using citrated human plasma and sheep RBCs” section of the Materials and Methods. The fourth sentence of the second paragraph should read: After incubation, the reaction was quenched by the addition of 1 ml of VBS-EDTA buffer (2.5 mM barbital, 1.5 mM sodium barbital and 144 mM NaCl, and 2 mM EDTA, pH 7.4).

The sixth sentence of the second paragraph should read: Plasma, diluted with AP-CFTD buffer containing 50 mM EDTA was treated in the same manner and used as a blank.
