# Supplementary figures and images for: A Novel Quantitative Hemolytic Assay Coupled with Restriction Fragment Length Polymorphisms Analysis Enabled Early Diagnosis of Atypical Hemolytic Uremic Syndrome and Identified Unique Predisposing Mutations in Japan
Source: PLoS One. 2015 May 7;10(5):e0124655. doi: 10.1371/journal.pone.0124655 (PMC4423893; doi:10.1371/journal.pone.0124655)

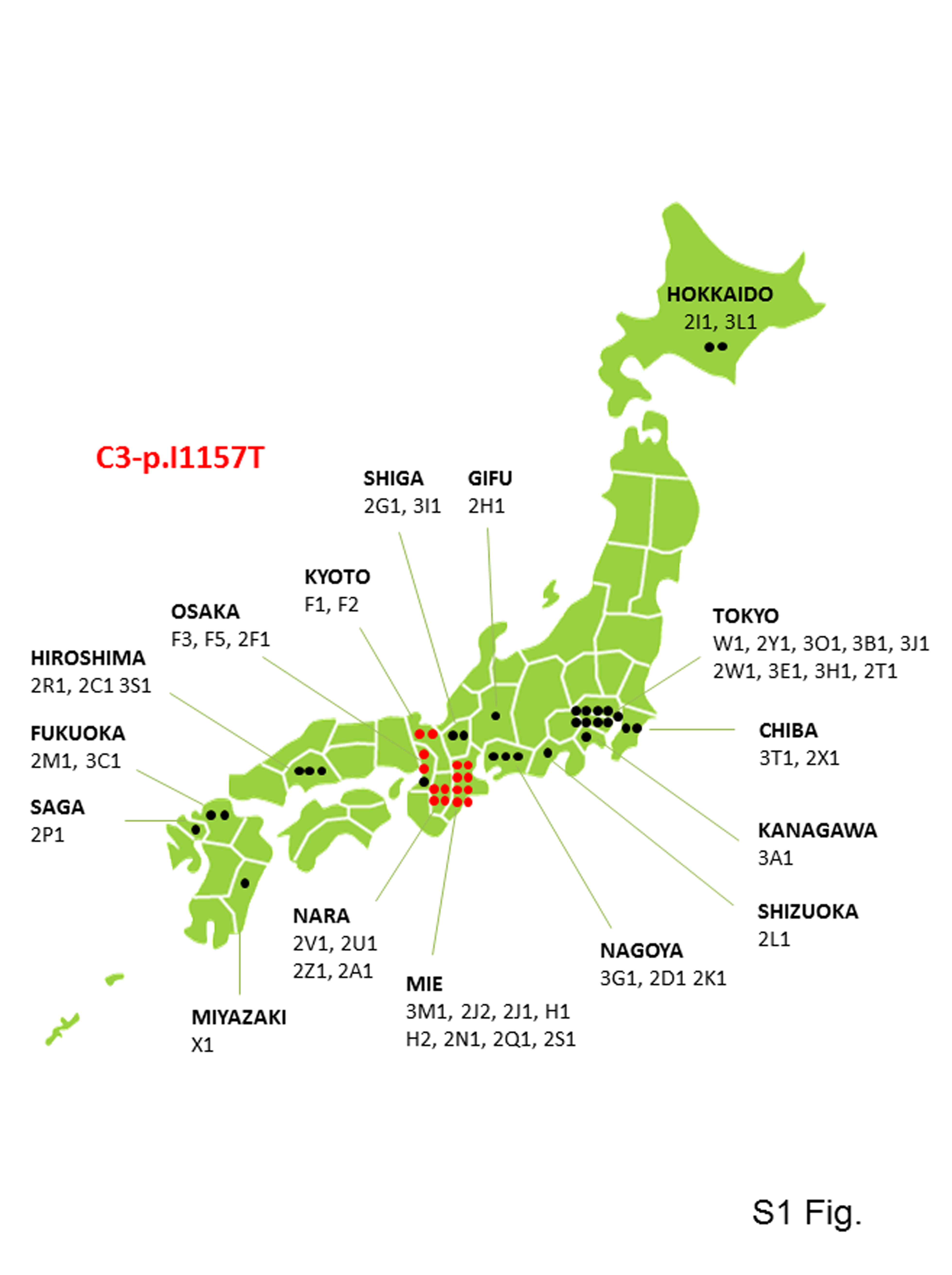

Supplement: S1 Fig — Each aHUS patient is described as a black or red circle. Sixteen patients with C3-p.I1157T mutation shown by red circles were found only in the Kansai district, including Mie, Nara, Kyoto, and Osaka prefectures. Interestingly, patients carrying C3-p.I1157T mutation were not found in other areas of West Japan, which may indicate a reflection of ‘founder effect’. (TIF) [file pone.0124655.s001.tif]

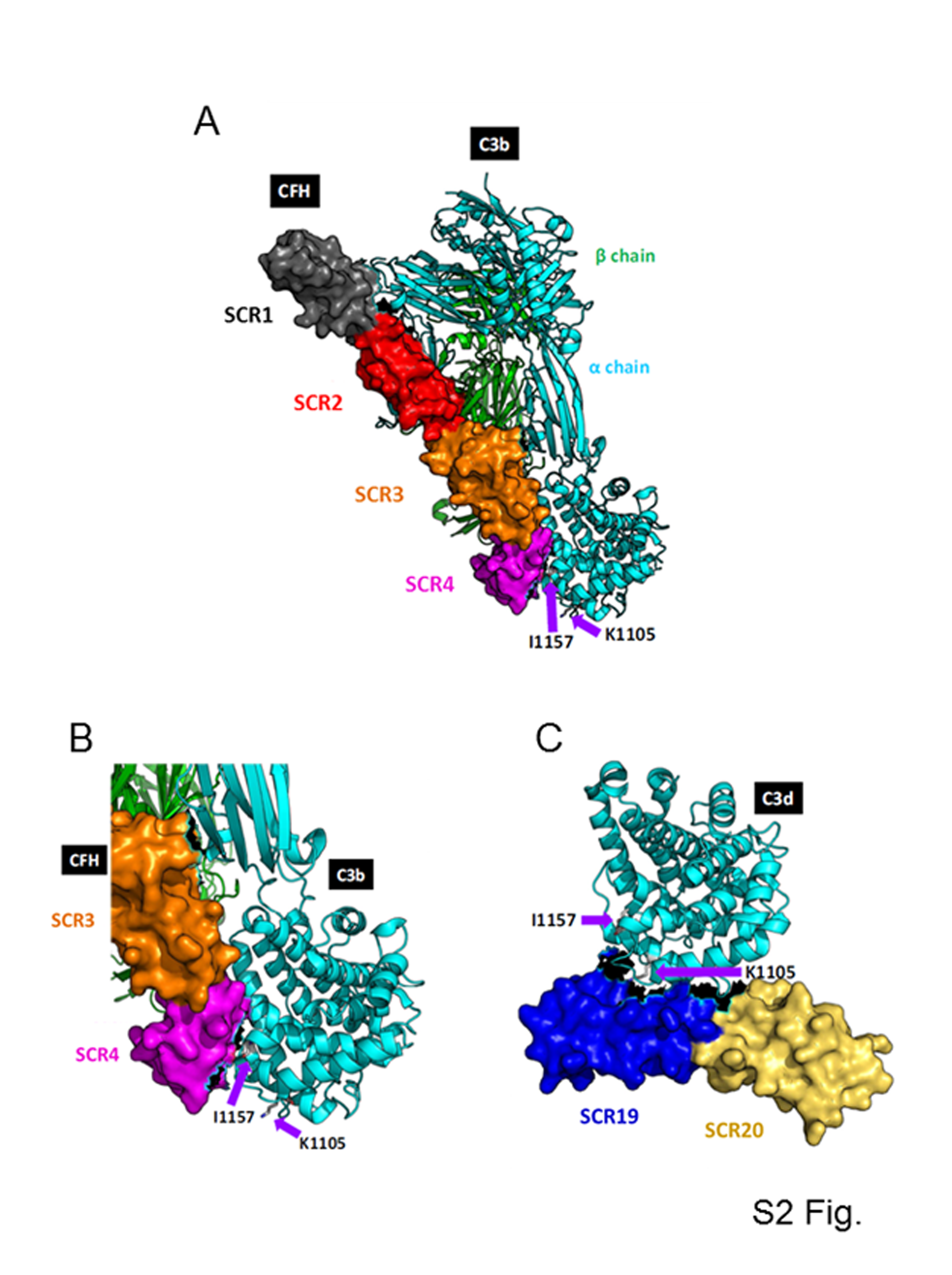

Supplement: S2 Fig — (A) A structural model of the complex of C3b and short consensus repeat (SCR) 1–4 of CFH (ID: 2WII). The α and β chains of C3b are shown in cyan and blue, respectively and the SCR1-4 domains are depicted with gray, red, orange, and magenta, respectively. The I1157 and K1105 residues shown by the grey-labeled side chains are located in the thioester-containing domain (TED) in C3b. (B) Close-up view of the region around the contact interface between C3b and SCR1-4. The p.I1157T mutation identified in 16 patients with low (less than 50%) hemolytic activity, but not the p.K1105Q mutation identified in a patient with high (100%) hemolytic activity, is positioned at the interface between C3b and SCR1-4 and would interfere the C3b binding to CFH-SCR1-4. (C) A structural model of the complex of C3d and SCR19-20 of CFH (ID: 3OXU). C3d, SCR19, and SCR20 are depicted with cyan, blue, and yellow, respectively. The p.K1105Q mutation, but not the p.I1157T mutation, is positioned at the interface between C3d and SCR19-20, and would interfere the C3b binding to CFH-SCR19-20. Diagram was generated with the PyMOL molecular visualization system. (TIF) [file pone.0124655.s002.tif]

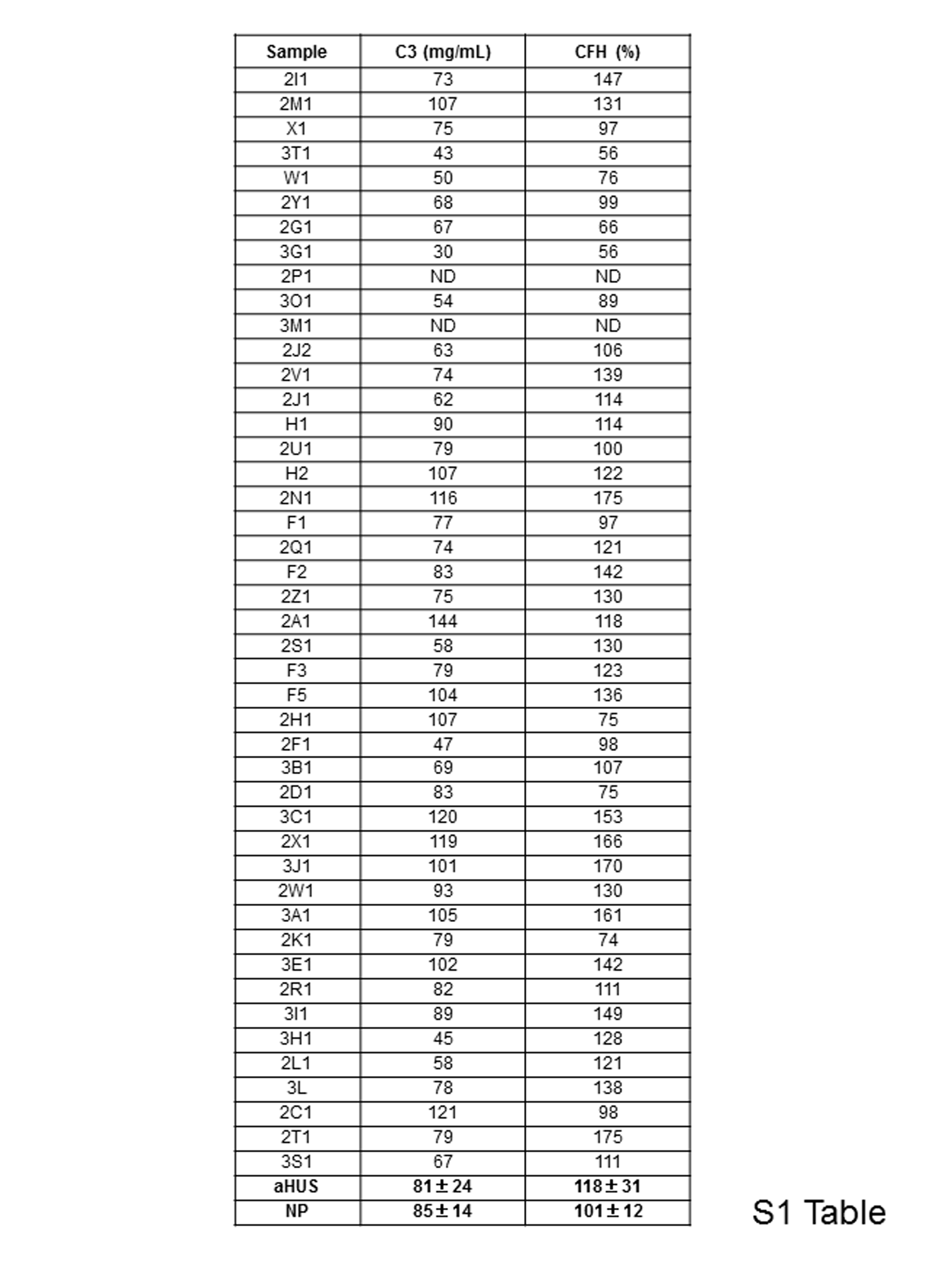

Supplement: S1 Table — C3 level was determined by immune-nephelometry (SRL, Inc., Japan), and CFH level was measured by Laurell’s immunoelectrophoresis using rabbit anti-CFH serum prepared in our laboratory. The levels of C3 and CFH in 43 aHUS patients and the mean ± standard deviation of these patients and normal plasma from 20 healthy individuals were described. CFH: complement factor H, C3: complement component C3, NP: normal plasma, ND: not determined (TIF) [file pone.0124655.s003.tif]

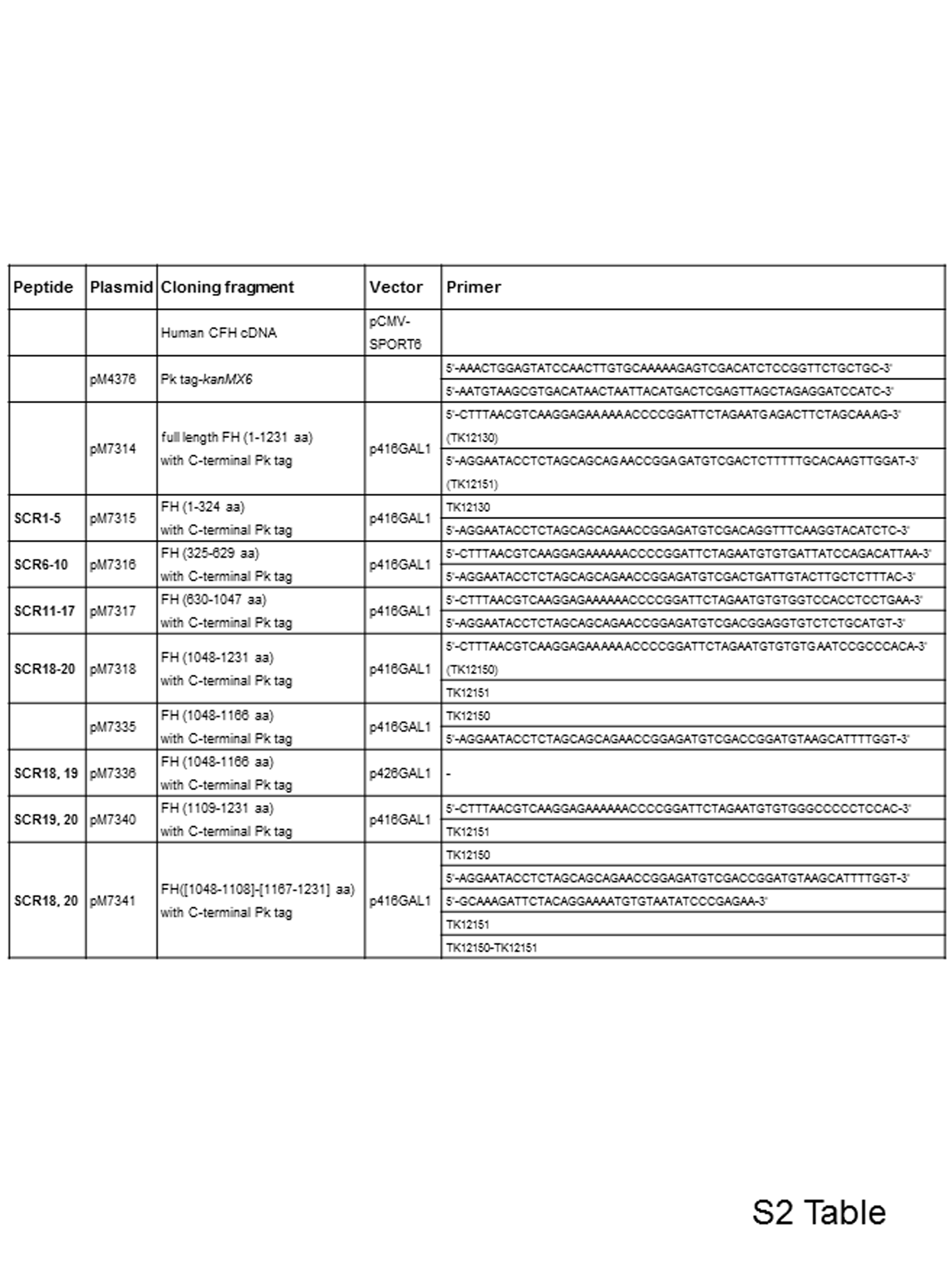

Supplement: S2 Table — SCR: short consensus repeat, CFH: complement factor H, Pk tag: GKPIPNPLLGLDST sequence, aa: amino acids. (TIF) [file pone.0124655.s004.tif]

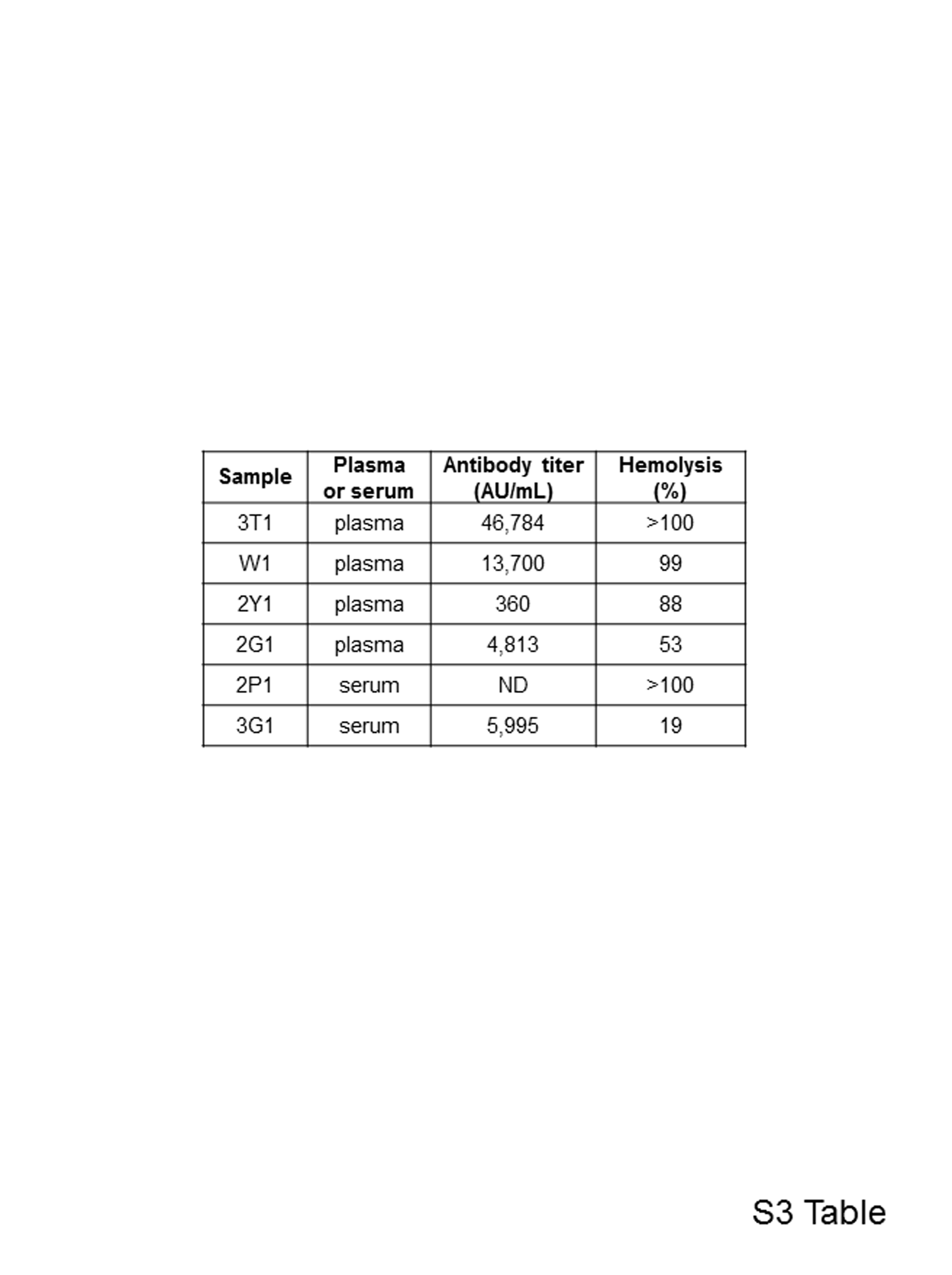

Supplement: S3 Table — Determination of CFH autoantibody titer was performed by CFH-IgG ELISA kit (Abnova). Antibody titer was calculated according to the manufacturer’s protocol by using standard curve. AU: arbitrary unit, ND: not determined. (TIF) [file pone.0124655.s005.tif]
